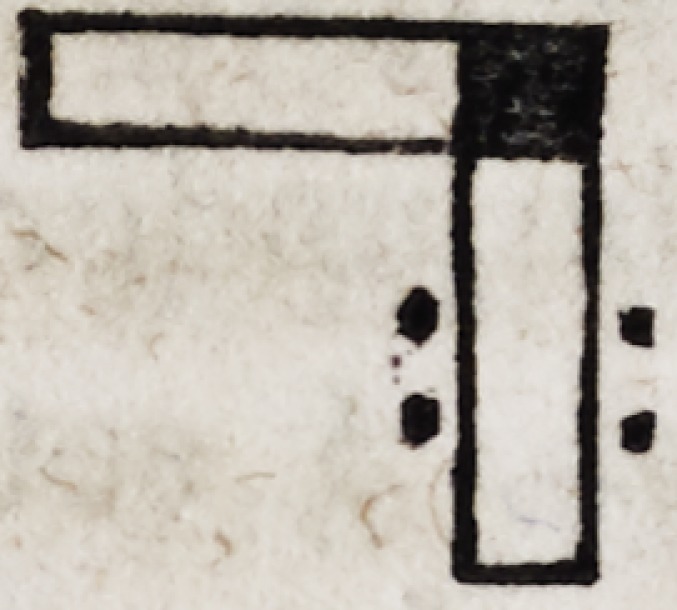# A Remedy for the Protrusion of the Lower Jaw

**Published:** 1842-09

**Authors:** J. S. Gunnell

**Affiliations:** Washington City, D. C.


### ARTICLE III.

### *A Remedy for the Protrusion of the Lower Jaw.* By J. S. GUNNELL, M. D., Washington City, D. C.

ABOUT the year 1822 or 1823, my attention was called to the necessity of some plan, whereby the protrusion of the lower jaw (called jimber or morose jaw) should be restored to its proper situation so that the under teeth (in front) would close under and posterior to the upper ones; thereby forming or restoring the natural appearance of the human mouth. I found in all such cases, that by the strongest sufferable pressure with the hand on the chin, the jaw could not be pressed back so as to bring the under teeth into their natural relative position to the upper ones. I set about discovering some remedy more convenient and expeditious than any then known.

In a conversation I had with Dr. H. H. Hayden, about the above period, he advised pressure by straps, extending from the chin to the back part of the head, &c. but finding them very difficult to retain in place, I determined to use Mr. Joseph Fox's* bandage, or cap and straps, which he used for the prevention of the sudden luxation of the lower jaw from gaping.

The protrusion of the lower jaw, or natural partial luxation, if I may use the expression, sometimes occurs from nature's imperfect operation, but seldom takes place before the cutting of the second set of teeth, though I have seen it exist in several cases previously to that period.† There is a defect of the kind in the jaw of the little daughter of Mr. Walter H. S. Taylor, of George-

* See plate ix. figure 5, in Mr. Joseph Fox's work on the human teeth, &c.

† In conversation with one of the editors, Dr. Gunnell explained the probable cause of jimber-jaw at this early age in some of the cases which occur, although it is no doubt in many cases hereditary. It is known that the incisors of the lower jaw are first cut, so that when the upper ones make their appearance, the lower have nearly arrived at their full growth. Of course in closing the mouth they come in contact with the gum on the inside of the upper incisors, and for relief the little sufferer thrusts forward the under jaw, which by long use becomes permanent; causing the deformity proposed to be remedied above. We would recommend the plan above proposed, having met with entire success in several cases in which we tried it by Dr. G's advice. M.

town, of this District; the other cases were restored in the way to be described below.

But the most common time for the occurrence of the protrusion of the lower jaw is soon after the commencement of second dentition. It is caused at this period by the teeth coming out irregularly, so that the teeth of the upper jaw strike upon or just inside of the edge of those of the lower. The consequence is, that when the jaws are brought together, the lower jaw is forced forward, producing its partial luxation at the tempero-maxillary joint. It will be entirely prevented by timely extraction of the infant teeth, and pressing the adult teeth in their proper places, &c.

To restore the jaw thus deformed, I proceed by tying a small block of ivory on one of the lower jaw teeth, so as to separate the teeth in front about one-quarter or one-eighth of an inch, and then put on Fox's bandage, and buckle or draw it as tight as the patient can bear with convenience, which produces pressure on the chin upwards and backwards; and then in case the teeth are irregular, take a piece of tough wood of the shape of a narrow spoon handle, introduce it between the teeth, and press it on the outside of the front lower protruding tooth or teeth, and on the inside of the upper irregular teeth, firmly, for from five to ten minutes, two or three times a day, the lower end of the stick or piece of wood and hand being below the chin, thereby pressing the lower teeth inwards and backwards, and the upper teeth outwards and forwards. In this way I have restored the face or jaws to their proper symmetry in one week, though occasionally it will take from three to six weeks or even longer.

When there are irregularities of the teeth in the case under operation, the remedies for such irregularities should go on at the same time with the above operations.

You will perceive that I treat this case somewhat as a luxation of the joint from any cause (and in this particular, differs from the common irregularity of the teeth alone; though both cases frequently exist together) as I consider it generally to be produced by a gradual partial luxation of the joint by the protrusion forward of the lower jaw, by an improper or unnatural pressure of the under and upper front teeth, &c. against each other, which generally commences in such cases about the time of cutting the second set or adult front teeth.

The operation of the bandage or cap and straps and block of ivory, is to press the joint ends of the lower jaw backwards and downwards, and press the chin backwards and upwards, the block of ivory between the back teeth or molares acting as a fulcrum

This operation is best performed so soon as the deformity occurs, though it is performed with great certainty until puberty. And I have restored it much later, but the difficulty increases very much after the patient is sixteen years of age.

If the patient goes to school, the bandage may be taken off during the time of recitation, though if kept off long it will protract the restoration. I generally advise that the bandage should be removed only to comb the hair, &c. about a half hour or an hour every morning and evening, and the face washed and rubbed freely, to remove constraint of the jaws: where the skin becomes sore from the pressure of the strap or bandage which should be made tighter every day or two, there should be a soft pad put on the chin to prevent friction.

The cure may be considered complete when upon removing the block of ivory, the lower jaw is found to close in its proper position, the teeth in front being posterior to those of the upper. It may be well, however, to use the bandage during the hours of sleep and meals for a short time. I have cured a considerable number of cases in this way and recommend it with great confidence.

## Figures and Tables

**Figure f1:**